# Exfoliative Endometrial Cytology in Embryo Donor Cows—Comparison of Sampling Localizations for the Diagnosis of Subclinical Endometritis

**DOI:** 10.3390/vetsci3040035

**Published:** 2016-11-28

**Authors:** Janna Egberts, Jan Detterer, Arno Park, Sabine Meinecke-Tillmann

**Affiliations:** 1 AI- and ET-Center Georgsheil, VOST, Am Bahndamm 4, Südbrookmerland 26624, Germany; j.detterer@vost.de (J.D.); a.park@vost.de (A.P.); 2 Institute for Reproductive Biology, University of Veterinary Medicine Hannover, Bünteweg 2, Hannover 30559, Germany; sabine.meinecke-tillmann@tiho-hannover.de

**Keywords:** exfoliative cytology, cytobrush, subclinical endometritis, dairy cows, embryo donor cows, PMN

## Abstract

Subclinical endometritis has a major effect on the reproductive performance of dairy cows and also on the success of embryo collection. Thus it is important to minimize the number of false-negative diagnoses. In order to evaluate the question of whether or not a single cytobrush sample is representative of the whole endometrium, 53 German Holstein embryo donor cows in the northwest of Germany were examined via the cytobrush method at three different localizations of the uterus: the uterine body about 0.5 cm cranial of the cervical canal and both uterine horns about 1.5 cm cranial of the bifurcation. Although the mean percentage of polymorphonuclear neutrophils at the three locations is not significantly different (*p* = 0.64), the individual variations lead to the conclusion that more than one sample of the endometrium should be taken into account when diagnosing subclinical endometritis in embryo donor cows.

## 1. Introduction

Subclinical endometritis (SE) is defined as a postpartum inflammation of the endometrium in the absence of any signs of clinical endometritis such as purulent vaginal discharge [1]. In order to diagnose SE, the proportion of polymorphonuclear neutrophils (PMN) in endometrial samples is evaluated.

Endometrial cytology has been proven to be a reliable method to detect SE, whereas bacteriological testing is not recommended since SE seems to be more of a problem of the recovery of the uterus postpartum than an infection [1,2,3,4,5,6,7]. Due to the impact of estrus cyclity, including the infiltration of PMN, which is lower on superficial compared to deeper layers of the endometrium, uterine biopsies are more sensitive to physiological changes than endometrial cytology [8,9]. Hence it is recognized that there is low correlation between endometrial biopsies and exfoliative cytology for diagnosing uterine disease [5].

Although there is no gold standard for the diagnosis of SE, cytobrushing has been used in the majority of studies and has been considered to be reliable, quick and easy [2,4,5,6,9,10,11] in contrast to low-volume flush [3,12]. Samples are taken from the most superficial layers of the endometrium, which makes cytobrushing insensitive to changes during the estrus cycle [5], which results in a reduction of false-positive outcomes. However, so far, and without regard to the possibility of localized events, cytobrush samples are mainly taken from a single intrauterine localization, based on the assumption that the diagnostic conditions would not differ throughout the whole endometrium, which has been reported for endometrial biopsies [13].

SE in cattle affects the reproductive performance as far as increasing the interval between calving and conception by about 30 days and decreasing the pregnancy rate by about 16% [1,3,4,5]. This results in major economic losses. An influence on the success of embryo collection has also been reported, where more unfertilized oocytes and fewer transferable embryos of poorer quality are collected from cows suffering from SE compared to healthy donor cows [10,11].

To allow for the proper management of those animals and to improve the success of embryo collection, it is important that the number of false-negative diagnoses is minimized.

The aim of this study was to answer the question of whether a cytological smear preparation taken from a single intrauterine localization is representative of the whole endometrium.

## 2. Materials and Methods

Fifty-three German Holstein cows in their first to tenth lactation (4.3 ± 1.8 lactations/animal; means ± SD) were investigated between D 64 and 1018 postpartum (D 215 ± 165 p.p.; means ± SD). The animals (client-owned: informed client-consent) were presented as embryo donors in a commercial embryo transfer program. Before superovulation, all participants were clinically examined during interoestrus (D 6–13 of the oestrus cycle). With regard to standard transrectal palpation and ultrasound scanning (Easi-Scan^®^, BCF™ Technology, broadband straight linear rectal probe, 7.5 MHz) of the reproductive tract as well as vaginoscopic inspection of the vagina and external os of the cervix showed no signs of clinical illness.

Exfoliative cytology samples were obtained with commercial cytobrushes (Gynobrush^®^, Heinz Herenz Medizinalbedarf GmbH, Hamburg, Germany), which had been attached to a universal A.I. gun without mandrin (Kombicolor, IMV, L’Aigle, France). These extended cytobrushes had a total length of 54 cm. In order to expose only the head of the cytobrush to the desired location of the endometrium, sterile casing aluminum pipes (47 cm long, outer diameter: 6 mm, inner diameter: 4 mm) with rounded tips were used to pass the cervix and to reach the desired intrauterine locations under transrectal control. To protect the sample from vaginal contamination and to ensure a clean sampling system to pass the cervix the whole system was placed in an A.I. sanitary sheath (IMV, L’Aigle, France), which was only perforated directly in front of the external os of the cervix.

Exfoliative cytology samples were taken by two experienced examiners from three different uterine localizations per animal, including both uterine horns about 1.5 cm cranial of the bifurcation and the uterine body about 0.5 cm cranial of the internal os of the cervix (standardized conditions). These localizations were chosen referring to the regions of interest in an ultrasonic echotexture study of the bovine uterus [14]. Sampling started in the uterine body (UB), followed by the right (RUH) and left uterine horn (LUH), each time with a fresh sampling system. The obtained cellular material was spread out on microscope slides, air dried, fixed, stained with DiffQuick^®^ and covered with a HistoKitt^®^ fixed cover glass. A total of 300 cells/slide were differentiated microscopically (light microscope, 400× magnification, Olympus, BH-2, Tokyo, Japan) by a single skilled investigator and the percentage of PMN was assessed [6]. The threshold level of PMN indicating SE was set at 5% [6,15]. A total of 159 microscopic slides were investigated.

All results are displayed with means ± SD. Statistical analysis was done using One Way Analysis of Variance on Ranks with InStat3 (GraphPad Software, Inc., La Jolla, California, USA).

## 3. Results

The mean percentage of PMN found throughout all differentiated smears was 2.4% ± 1.7%. Although statistically not significant (*p* = 0.64), the mean percentage of PMN found in the uterine body (2.2% ± 1.8%) was generally lower than in the right (2.3% ± 1.5%) and left uterine horns (2.5% ± 1.7%).

In 13 smears the PMN threshold of 5% was exceeded, and 18.9% (10/53) of the cows were diagnosed with SE. Intra-individual differences became obvious, yet in each one of these SE-positive animals, only one or two of the samples exceeded the threshold level for SE. In two individuals SE was verified in both uterine horns (individual results: UB: 4% and 1.4% PMN; RUH: 6.4% and 5% PMN; LUH: 5.7% and 5% PMN), the third surpassed the threshold in the uterine body as well as in the right uterine horn (individual results: UB: 7.7% PMN; RUH: 5.4% PMN; LUH: 1.4% PMN) and the other seven animals were diagnosed with SE only in the right (individual results: UB: 3.7% and 3% PMN; RUH: 5.4% and 5.4% PMN; LUH: 4.4% and 4% PMN) or left uterine horn (individual results: UB: 0.7%, 2.7% and 2.0% PMN; RUH: 4%, 3% and 3.4% PMN; LUH: 5%, 6.7% and 5% PMN) or the uterine body (individual results: UB: 5% and 6.7% PMN; RUH: 0.4% and 3% PMN; LUH: 4.7% and 4.4% PMN).

## 4. Conclusions

The results demonstrate that it might be advisable, for safe diagnosis of subclinical endometritis in embryo donor cows or cows well past the postpartum period, to take at least two cytological samples from different intrauterine localizations, preferably the uterine horns. The cow should be seen as positive for SE if at least one sample exceeds the threshold level of 5% PMN. A single exfoliative cytology does not seem to represent the entire endometrium. These findings might also be an argument to investigate this problem further using the low-volume flush technique, where a larger region of the endometrium is sampled.

## Abbreviations

The following abbreviations are used in this manuscript:

SE: subclinical endometritis
PMN: polymorphonuclear neutrophils
UB: uterine body
RUH: right uterine horn
LUH: left uterine horn
